# Deploying Machine Learning Models Using Progressive Web Applications: Implementation Using a Neural Network Prediction Model for Pneumonia Related Child Mortality in The Gambia

**DOI:** 10.3389/fpubh.2021.772620

**Published:** 2022-02-18

**Authors:** Nuredin I. Mohammed, Alexander Jarde, Grant Mackenzie, Umberto D'Alessandro, David Jeffries

**Affiliations:** Medical Research Council Unit, The Gambia at the London School of Hygiene & Tropical Medicine, Banjul, Gambia

**Keywords:** artificial intelligence (AI), progressive web applications (PWAs), digital health, pneumonia, mortality, machine learning (ML)

## Abstract

**Background:**

Translating research outputs into practical tools for medical practitioners is a neglected area and could have a substantial impact. One of the barriers to implementing artificial intelligence (AI) and machine learning (ML) applications is their practical deployment in the field. Traditional web-based (i.e., server sided) applications are dependent on reliable internet connections, which may not be readily available in rural areas. Native mobile apps require device specific programming skills as well as contemporary hardware and software, with often rapid and unpredictable platform specific changes. This is a major challenge for using AI/ML tools in resource-limited settings.

**Methods:**

An emerging technology, progressive web applications (PWAs), first introduced by Google in 2015, offers an opportunity to overcome the challenges of deploying bespoke AI/ML systems. The same PWA code can be implemented across all desktop platforms, iOS and Android phones and tablets. In addition to platform independence, a PWA can be designed to be primarily offline.

**Results:**

We demonstrate how a neural network-based pneumonia mortality prediction triage tool was migrated from a typical academic framework (paper and web-based prototype) to a tool that can be used offline on any mobile phone—the most convenient deployment vehicle. After an initial online connection to download the software, the application runs entirely offline, reading data from cached memory, and running code *via* JavaScript. On mobile devices the application is installed as a native app, without the inconvenience of platform specific code through manufacturer code stores.

**Discussion:**

We show that an ML application can be deployed as a platform independent offline PWA using a pneumonia-related child mortality prediction tool as an example. The aim of this tool was to assist clinical staff in triaging children for hospital admission, by predicting their risk of death. PWAs function seamlessly when their host devices lose internet connectivity, making them ideal for e-health apps that can help improve health and save lives in resource-limited settings in line with the UN Sustainable Development Goal 3 (SDG3).

## Background

Research on the application of machine learning algorithms in the healthcare sector has grown quickly in recent years and aims to develop and improve disease diagnosis, prognosis, and treatment, including personalized medicine (1). This in turn can accelerate the attainment of Sustainable Development Goal 3 (SDG3).^1^ However, the deployment of such models is challenging in resource-limited settings, particularly in peripheral health facilities. For example, biomarkers are widely used in clinical medicine in several prediction frameworks, but their publication in the scientific literature rarely results in marketable applications (2). Two key impediments to successfully deploying machine learning (ML) based prediction models are the lack of standardization of research methods (3–5) and lack of engagement from practitioners.

We aim to demonstrate a generic deployment process for neural networks using, as an example, a recent pneumonia mortality prediction machine learning algorithm (6) developed at the Medical Research Council Unit The Gambia at the London school of Hygiene and tropical Medicine (MRC Unit The Gambia at LSHTM). The initial goal of this study was to develop and validate a predictive mortality model in children 1–59 months old using variables readily available to clinicians at the time of admission. As the vast majority of childhood Pneumonia deaths occur in low and middle income countries (7), it is important health workers can easily access and implement these predictive models in their clinical practice. This means deploying a prospective tool across all mobile platforms with seamless offline functionality as health facilities may have compromised internet connectivity. The primary aim of this paper is to describe the deployment of a neural network model; a complete description of the machine learning algorithm is given in another publication (6).

The machine learning model was developed using R software (8) as it is a fast development environment and widely used language for statistics and data science. The tool^2^ was initially deployed as a web application using R Shiny^3^ as it can then be conveniently deployed and distributed, *via* a private webserver, GitHub (a software repository) or shinyapps.io (a public webserver). Shiny is a web framework for R allowing rapid prototyping and deployment for researchers and data scientists. It is a server sided application, with no offline capacity and webpages do not always integrate well with mobile devices.

The primary requirement for the deployment of our proposed tool was that it should be available offline, on standard tablets and phones (mainly Android with an iOS minority) used in The Gambia. Secondly, we wanted it to run as a native app, but avoid the development of product and version specific apps as they add time, cost and skills overheads.

Until 2015, there was little alternative to developing platform specific apps. However, this changed with a new set of standards for Progressive Web Apps (PWAs) published by the Google Web Framework (9). In addition to cross-platform app compatibility, these standards also introduced offline support, synchronization tools and automatic conversion to app icons, i.e., no sideloading.

## Methods

PWAs are multiplatform web application development approaches that can operate on most mobile and desktop platforms (Android, iOS, Windows, Linux.) *via* a browser which is ubiquitous on most operating systems (10). In addition to the usual web application components of HTML, CSS and JavaScript (JS), a PWA also consists of a manifest file and a service worker. The manifest file controls how the PWA is integrated into the desktop or mobile platform. A service worker is a JavaScript file that takes requests from the application and if it detects that the user is offline redirects requests to the appropriate cached resources for a seamless offline experience (11). PWAs are usually implemented *via* JavaScript and many web frameworks (software methods for standardizing and structuring web site development) have tools for bespoke development of PWA. For transparency, we wrote the code without using a web framework.

The original machine learning prediction model (6) was fitted to data on 11,012 children with clinical pneumonia from hospital admission data in The Gambia. The neural network was fitted in R using the caret machine learning library (12). To implement the machine learning component in a PWA, it must be converted into a JavaScript compatible format. The most efficient approach would be to transform the native R formats of the already fitted neural network directly into a JavaScript TensorFlow^4^ format, as it requires no recoding of the original model. Unfortunately, we were not able to convert the neural network weights and meta data into the necessary JavaScript TensorFlow format, but this remains a desirable goal.

TensorFlow is an open source machine learning library with an R interface and we amended the original R code to use TensorFlow with the Keras (13, 14)^5^ R library to fit a neural network to the same data as in Jarde et al. (6). We re-coded the nested cross-validation in the original R code using the TensorFlow library rather than the caret library. The TensorFlow/Keras R libraries have lower-level functionality than caret and there is no direct replacement for the convenient wrapper functions. As the wrapper functions tend to obfuscate the processes it was not a straightforward code substitution process to replicate the original model. The nested cross validation algorithm with its performance metrics are described in detail in the Appendix 1 in Supplementary Material.

The chosen Neural Network from the TensorFlow/Keras R code was saved in a native TensorFlow format. This format can be used across both R and Python, but not in web-based JavaScript application. The native format can be transformed into an appropriate JavaScript format using the TensorFlow JavaScript converter (for more details see the Appendix 2 in Supplementary Material).

## Results

With the neural network now available in a JavaScript compatible format, a standard HTML webpage can be built, where users can input patient data for the selected predictor variables. These are then imported to the neural network model *via* JavaScript code, that predicts the mortality risk. Even for clinical staff, risk probabilities may not always be easy to interpret, especially if the mortality threshold cut-off is not 0.5. We used the positive and negative likelihood ratio discrimination metrics to divide the risk into low, medium and high categories. (Appendix 3 in Supplementary Material). For a production version, it is likely that the risk stratification would also involve consultation with clinical staff and not be based purely on an algorithmic approach. The relevant risk category is then returned to the webpage *via* HTML. This is a standard web application, which becomes a PWA when a manifest and a service worker are included. As we are releasing a publicly available version of this PWA, we have anonymized the 5 predictor variables as variable 1 to 5 to prevent inappropriate use.

**Figure 1:**
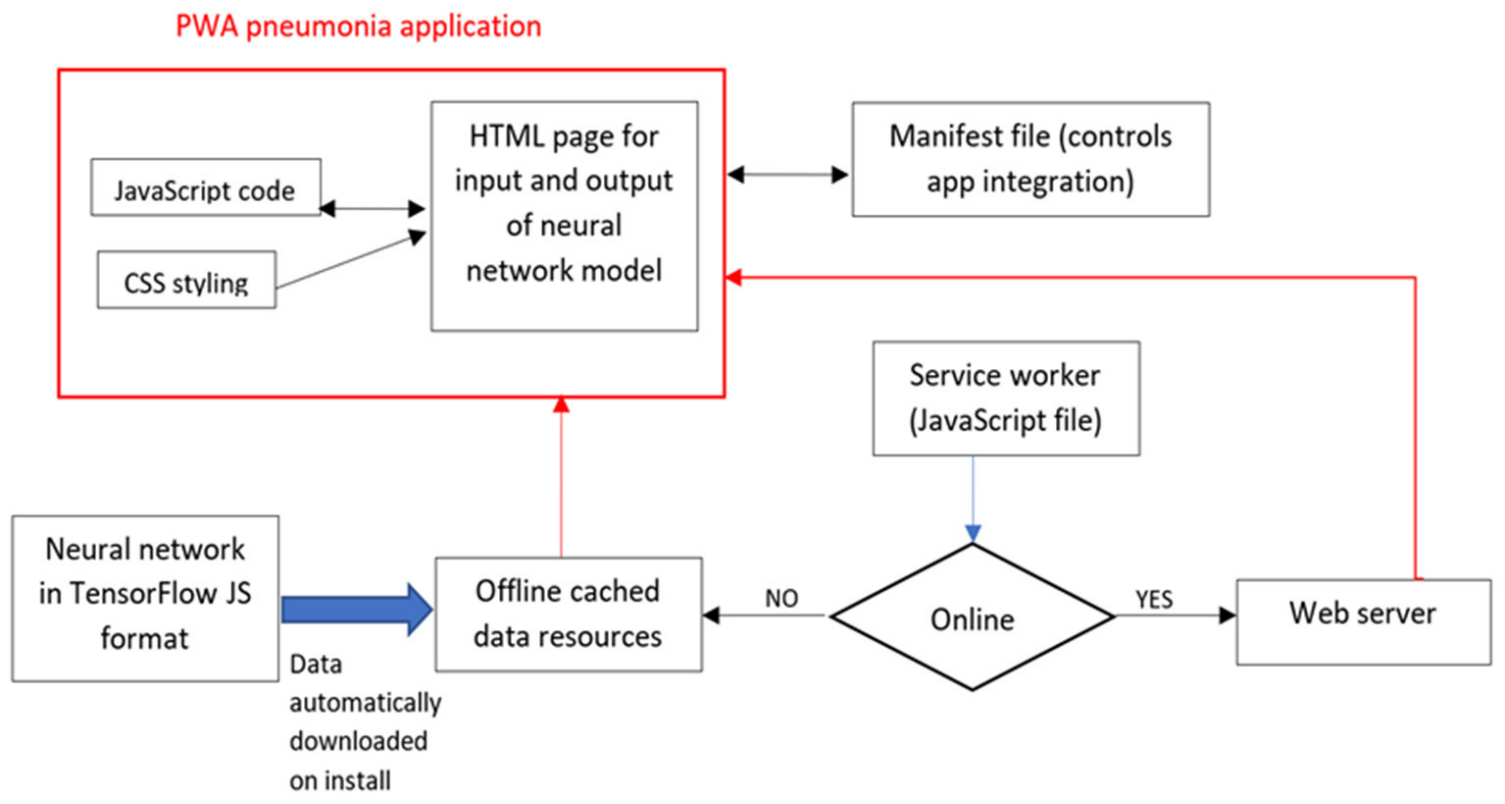
Structure of PWA for pneumonia prediction.

Figure 1 shows the structure of the application (described in more detail in the Appendix 4 in Supplementary Material) and the central role played by the service worker. Users can now access the PWA application from a web server, which are the hardware and software necessary to distribute webpages. These are extremely common and can be locally or cloud hosted and are straight forward to set-up. The code (CSS, JavaScript and HTML) are stored in the Cache Storage for offline use. Data (input and output) are more conveniently stored in an IndexedDB database, where each saved object has a key, easily allowing it to be set or retrieved *via* JavaScript code. Data stored here can also be encrypted, which is important for clinical data. Cache Storage and IndexedDB are available in all modern browsers. In this example the TensorFlow neural network meta data is stored in an IndexedDB and no patient data is stored.

**Figure 2:**
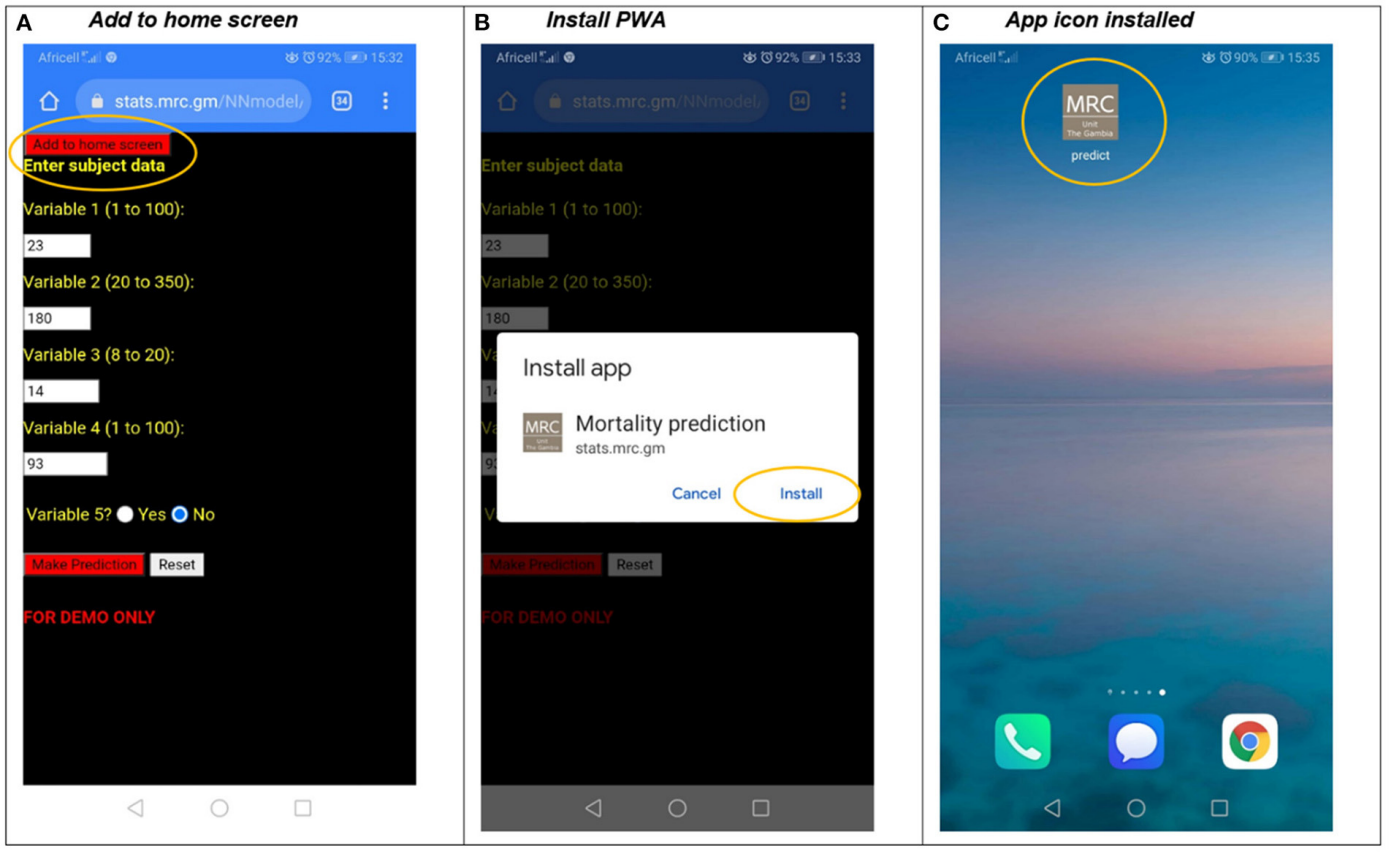
Installing a PWA application on an android phone.

The PWA pneumonia mortality prediction app is hosted on the MRC Gambia servers at *https://stats.mrc.gm/NNmodel*. Given that PWA was a Google initiative, Chrome gives the best browser experience, although it is also supported on all major browsers. When users connect to this application on an Android phone, they see the mortality prediction webpage, but with an “add to home screen” button at the top of the screen (this is controlled by the manifest file, described above in the previous section and in the Appendix 4 in Supplementary Material), as shown in Figure 2A. After clicking this button, users are prompted to install the webpage as PWA from an “Install app” pop-up message (Figure 2B), which creates the app icon (defined in the manifest file, Figure 2C).

**Figure 3:**
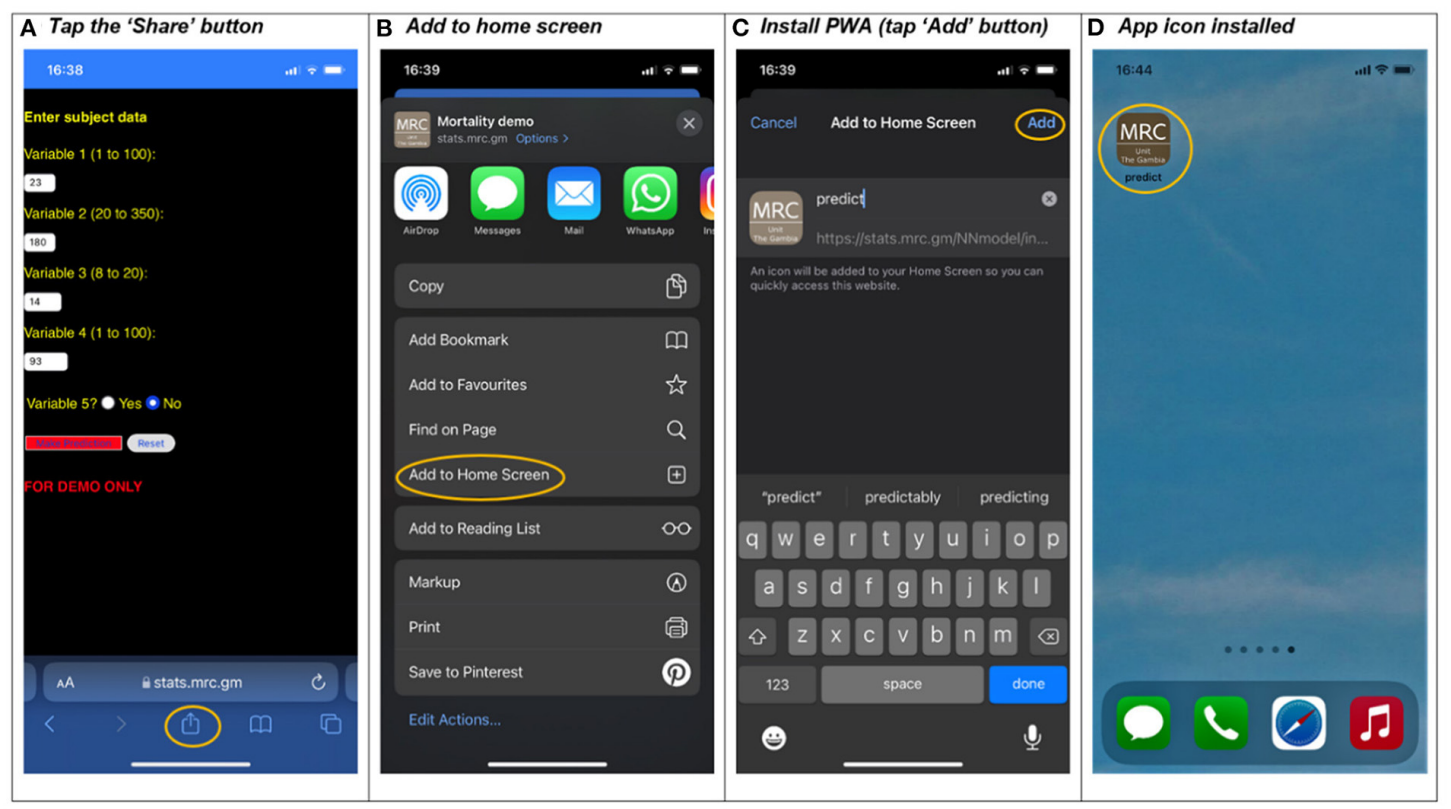
Installing a PWA application on an iOS phone.

The icon in Figure 2C can now be clicked and the prediction tool can be used like a “native app,” even though it is a webpage. As soon as the app has been downloaded it is immediately available to run as an offline app. The quality of the native experience depends on the styling of the webpage and web frameworks^6^ can provide a richer user experience in comparison to this vanilla application which has minimum styling. The integration of PWA with iOS is constantly evolving but offers a similar experience with PWA icon functionality, except that users have to click the iOS “Share” button and select “Add to Home Screen” from the list of options as shown in Figure 3. The most reliable and automated way to update PWAs with new content is to use push notifications, which are clickable messages that are propagated to appropriate users. They are accepted by default for Android users, but it is the opposite for iOS users and with rapidly evolving standards it is not currently a stable option for iOS users.

PWA applications also run on all major desktop browsers, but the integration available *via* the manifest file is constantly changing depending on browser policy. For example, since early 2021, desktop Firefox browsers have discontinued their support for installing a PWA as a standalone app. On all major desktop browsers, the service worker functionality allows the applications to run offline. The desktop browsers also offer the best facilities for debugging PWA code and users can examine the offline storage objects.

## Discussion

Health apps are generally deployed either as standalone (installed on a specific device) or web-based applications (via browsers). Standalone apps do not require an internet connection and can be distributed as executable code, written in programming languages such as C/C++, Java, R,Matlab, Python etc. These applications can be cumbersome to deploy to end users and requires the language and the application to be bundled into clickable apps using for example, Python's Pyinstaller (15), Matlab's compiler (16) or Electron based tools for R (17). Given the difficulties with these processes, Microsoft's Access and Excel with Visual Basic for Applications (VBA) (18), providing a comprehensive coding environment, are still widely used. However, VBA lacks the scientific libraries that are readily available in R, Python and Matlab. With these limitations, server sided web apps are an attractive alternative, and easy to deploy and update on any platform with an internet connection. The basic toolset of webpage design and functionality HTML/CSS/JS (19), has been complemented by many tools and frameworks that facilitate cross-platform production versions of scientific web-based apps such as Java Applets (20), Python's Django (21), R's Shiny and Matlab's web compiler (16). The primary disadvantage of these server-sided applications is that they require a reliable internet connection.

PWAs, *via* service workers allow seamless functioning of websites, even when the host device is offline. Additionally, they offer cross-platform deployment, and on phones and tablets the web-applications are run as “native-applications” from bespoke icons, without distribution through app stores. In a recent development, Microsoft has also embraced PWA technology *via* Blazor (22), which enables web development in C# rather than being restricted to JavaScript.

In this paper we have not discussed data synchronization, which is another feature available in a PWA. Using PouchDB on the client side (i.e., phone or tablet) and CouchDB on the server, synchronization between the two is automatic as users go on and offline,^7^ managed by the JavaScript service worker code. For programmers, this is a major advantage as there is no layer of code handling the synchronization between the client and server sided databases.

In this application we save no patient data but depending on ethical approval we could store the entered data and ask users to enter the actual mortality outcomes of anonymised subjects. This could provide a powerful central resource for pneumonia mortality machine learning prediction.

Generalization is a gold-standard for machine learning applications, but in situations such as this, different standards of care at different sites make this a very difficult goal. With easy deployment of machine learning applications, a centralized research institute could fit and deliver site specific models. Particular attention would be needed to avoid overfitting local data. PWAs on Android and iOS devices can access the GPS sensors with user permission and geolocation could restrict users to specific models for specific clinics. Data synchronization between the sites and the centralized location could enhance the performance of the learning algorithms.

Although offline usage is important for many settings, periodic use with very long offline periods could have unpredictable effects. Users will obviously miss app updates, but they will also be subject to the device operating system policy for managing app cache storage and inactive apps. There are also app storage limits, for example iOS currently limits the IndexedDB storage to 500 MB. We will explore using push notifications (23) to engage with active users and SMS for users who are offline.

Future work will also explore how to create PWAs for additional machine learning algorithms. R and Python are very common open-source programming languages for data science. They offer complete environments for training, validating, and testing machine learning algorithms. However, as demonstrated in this paper, there is no common standard for saving the model meta data. There are many JavaScript machine learning packages, but for most data scientists the pipeline of developing machine learning models that conform to good practice guidelines in JavaScript is daunting, lacking many of the validation and graphical tools offered by R and Python. Rapidly emerging technologies offer alternative languages to just using JavaScript in the web browser. For example, there is experimental work to develop a version of Python to run entirely in the browser.^8^

In summary, we have demonstrated how to develop a cross-platform machine learning app to implement a pneumonia mortality prediction model that can be used on or offline. Although applied to a particular model, the coding framework is generic and can be applied to many data science applications. Thus, we encourage researchers to consider PWAs for deploying data science based products, particularly those developing models to aid health workers in their daily efforts to improve health and save lives in low-income settings.

## Data Availability Statement

The data analyzed in this study is subject to the following licenses/restrictions: Please note the patient level hospital data is subject to ethical restrictions and is not publicly available. For access, please contact: Dr Grant Mackenzie Pediatrician/Clinical Epidemiologist MRC Unit the Gambia. Requests to access these datasets should be directed to gmackenzie@mrc.gm.

## Ethics Statement

The studies involving human participants were reviewed and approved by The MRC Gambia at LSHTM and Gambia Government Joint Ethics Committee, i.e., our paper is based on and used anonymized data from a previous study which was approved by this joint ethics committee. Written informed consent to participate in this study was provided by the participants' legal guardian/next of kin.

## Author Contributions

NM, AJ, GM, UD, and DJ contributed to the design and write-up of the study. DJ led the technical work converting the ML model into JavaScript compatible format assisted by NM. AJ, GM, and DJ developed the original model that was considered in this manuscript. All authors contributed to the article and approved the submitted version.

## Conflict of Interest

The authors declare that the research was conducted in the absence of any commercial or financial relationships that could be construed as a potential conflict of interest.

## Publisher's Note

All claims expressed in this article are solely those of the authors and do not necessarily represent those of their affiliated organizations, or those of the publisher, the editors and the reviewers. Any product that may be evaluated in this article, or claim that may be made by its manufacturer, is not guaranteed or endorsed by the publisher.

## Footnotes

^1^https://sdgs.un.org/goals/goal3. Goal 3 | Department of Economic and Social Affairs.

^2^https://github.com/MRCG-djeffries/mortality-prediction. GitHub - MRCG-djeffries/mortality-prediction.

^3^https://shiny.rstudio.com. Shiny.

^4^https://www.tensorflow.org/js. TensorFlow.js | Machine Learning for JavaScript Developers.

^5^https://tensorflow.rstudio.com/. TensorFlow for R.

^6^https://framework7.io/. Framework7 - Full Featured Framework For Building iOS, Android \& Desktop Apps.

^7^https://pouchdb.com/guides/replication.html. Replication.

^8^https://hacks.mozilla.org/2019/04/pyodide-bringing-the-scientific-python-stack-to-the-browser/. Pyodide: Bringing the scientific Python stack to the browser - Mozilla Hacks - the Web developer blog.
