# Supplementary material for: Deploying Machine Learning Models Using Progressive Web Applications: Implementation Using a Neural Network Prediction Model for Pneumonia Related Child Mortality in The Gambia
Source: Front Public Health. 2022 Feb 18;9:772620. doi: 10.3389/fpubh.2021.772620 (PMC8894647; doi:10.3389/fpubh.2021.772620)
Supplement: Supplementary file 1 [file Data_Sheet_1.pdf]

Supplementary material

Deploying Machine Learning Models Using Progressive Web Applications: Implementation Using a Neural Network Prediction Model for Pneumonia Related Child Mortality in The Gambia.

Nuredin I. Mohammed✉**, Alexander Jarde, Grant Mackenzie, Umberto D'Alessandro, David Jeffries**✉
Medical Research Council Unit The Gambia at the London School of Hygiene & Tropical Medicine, Banjul, The Gambia

✉Correspondence
Nuredin.Mohammed@lshtm.ac.uk
David.Jeffries @lshtm.ac.uk

All the code and data used to deliver the PWA application from https://stats.mrc.gm/NNmodel are available on the GitHub site, https://github.com/MRCG-djeffries/NNmodel, to implement on their own servers.

Appendix 1: Fitting neural network

The data set consisted of 11,012 children aged 1-59 months with clinical pneumonia. We split this dataset into a training and holdout test set, based on a chronological cut-off with 2/3 of the sample prior to July 16, 2015 comprising the training set. This resulted in a training set with 7,341 children with 167 deaths and a holdout test set of 3,671 subjects with 55 deaths. As the data are clearly imbalanced, we used a synthetic minority over sampling technique (SMOTE) to balance the training data.

We used nested cross validation to perform a grid search for tuning the learning rate and the number of nodes in the single hidden layer. The neural network was optimized using the RMSprop algorithm. During the neural network fitting three algorithms were used to reduce the chances of overfitting:

1. Hold out 30% of training set for validation during training
2. Node dropout rate of 30%
3. An early stopping patience of 5 epochs

The cross-validation process is shown in Figure 1. An outer loop repeated the procedure 1,000 times to obtain mean values and confidence intervals for the classification performance metrics.

After fitting 1,000 models we matched a model with an identical confusion matrix (shown below) based on the holdout test dataset, that resulted from a median performing neural network model in the original paper.

	**Predicted Alive**	**Predicted Dead**
**Actual Alive**	2779	837
**Actual Dead**	10	45

In the original paper the neural network was fitted using caret in R. For this paper the algorithm was converted to use the R TensorFlow (TF) and Keras libraries. Although the confusion matrices of the two selected models were identical, the predicted mortality probabilities were not the same, but agreement was very high (Lin's concordance 0.943, with 95% CI of 0.940 to 0.947). The Bland Altman plot for the two

sets of mortality probability predictions is shown in Figure 2. The discordance based on the differing optimum thresholds is shown below.

	**TF Alive**	**TF Dead**
**Caret Alive**	2716	72
**Caret Dead**	73	810

Given the differing algorithms, seeding and over-fitting protections in caret and TensorFlow it is unsurprising that the prediction probabilities are not in perfect agreement and not relevant to this paper, where the focus is on implementation. Table 1 shows the key performance metrics.

Figure 1: Nested cross validation algorithm

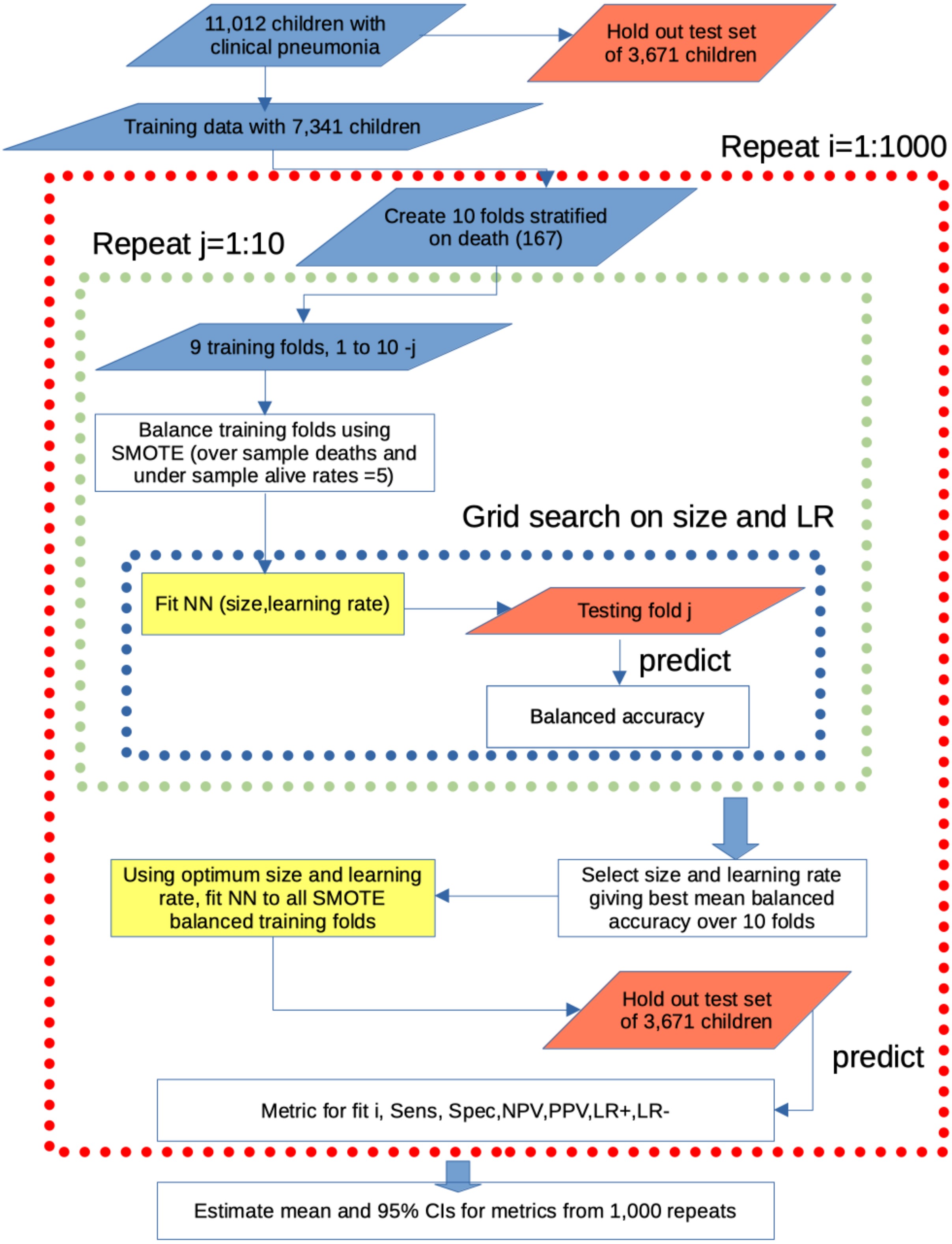

Figure 2: Agreement of mortality prediction from caret versus TensorFlow

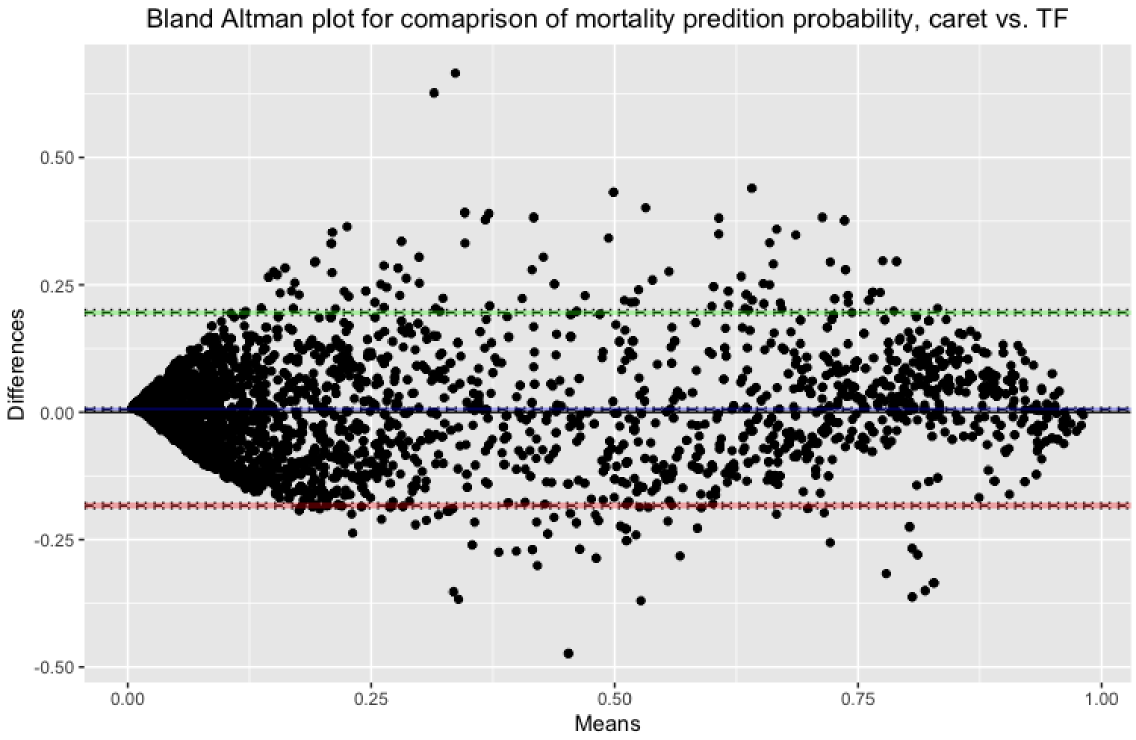

Table 1: Model performance metrics

Metric	Mean	2.5%	97.5%
Positive Predictive Value	5.3%	4.5%	6.3%
Negative Predictive Value	99.6%	99.5%	99.8%
Sensitivity	80.9%	70.9%	89.1%
Specificity	77.6%	71.8%	83.4%
Balanced accuracy	79.3%	76.0%	81.6%
Likelihood Ratio+	3.64	3.08	4.45
Likelihood Ratio-	0.25	0.15	0.35

Appendix 2: Exporting the chosen neural network model

The selected neural network from Appendix I was then saved to HDF5 format (hierarchical data format files), using the command save_model_hdf5, from the keras R library.

To convert this into a tensor flow JavaScript format (note test.h5 is the file created with the above command), within a terminal window type:

```
pip install tensorflowjs[wizard]
tensorflowjs_converter --input_format=keras test.h5 tfjs_model
```

The folder tfjs_model, now contains a JSON and binary file containing the meta and weight data of the neural network. This must be exported to the PWA file structure as shown below in Appendix 4.

Appendix 3: Mortality risk categories

The output of the tool is designed to give three risk categories (low, medium and high), which are easier for clinical staff to interpret than risk probabilities, especially when the threshold probability cut-off is not at 0.5. We determined these cut-offs, by quantizing the LR+ and LR- values against probability of death (see figure 3 below). In reality, it's likely that extensive consultation with clinicians would be required to extend this to care pathway recommendations.

Figure 3: Positive and negative likelihood ratio and cut-off probabilities for risk categories

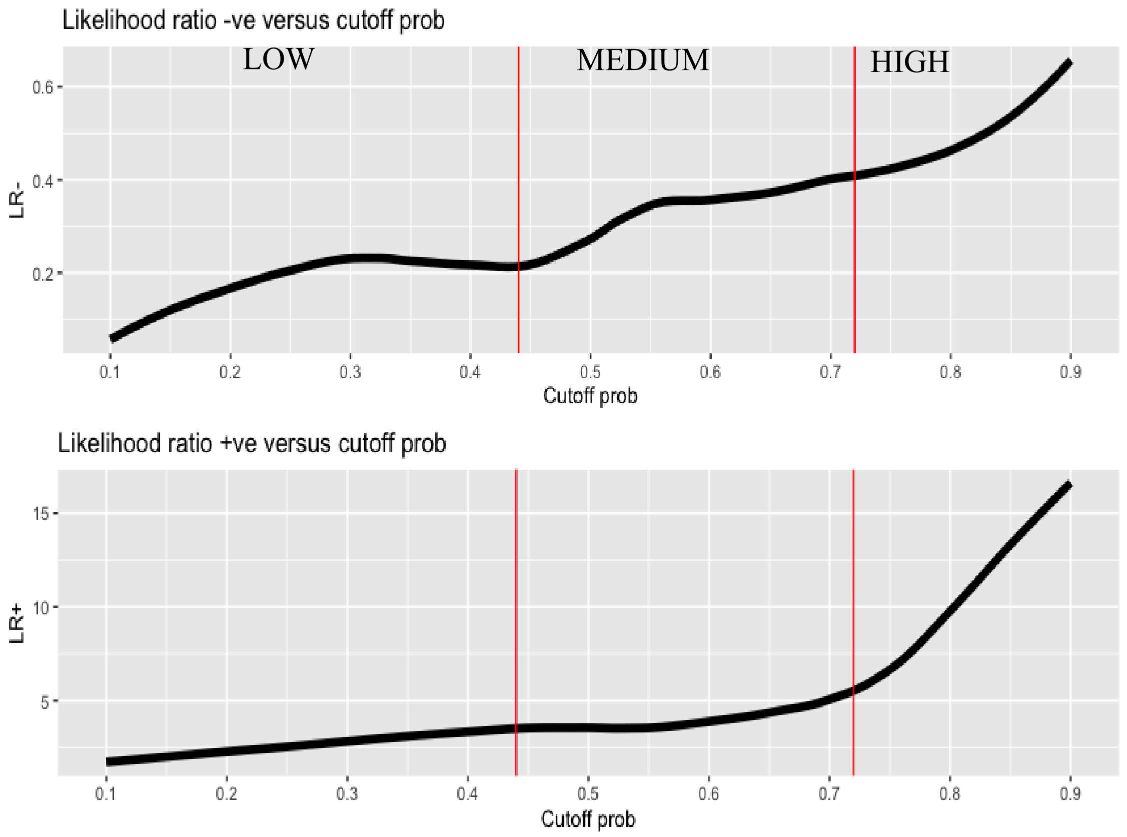

Appendix 4: Constructing the PWA application

The major components of a PWA application are:

- Web manifest file (JSON file)
- Service worker
- Usual JavaScript, CSS and HTML structure for a website.

The Web manifest contains the application meta data. The service worker manages the offline functionality and pushes data and libraries to off-line storage. Additionally for this application the TensorFlow JavaScript libraries should also be downloaded for this application. Also the neural network meta data (two files) also need to be included in the file structure.

The PWA application is stored in the repository Nnmodel on the GitHub page, https://github.com/MRCG-djeffries/Nnmodel. The folder contains the following files:

File	Description
index.html	Web page components
index.js	
*.css	HTML styling files
manifest.webmanifest	PWA components
sw.js	
tf.min.js	Tensor flow JavaScript library
tf.min.js.map	
Folder tfjs_model	Contains two files **group1-shard1of1.bin** and **model.json**, which contain the neural network weights and meta data, created in the previous section
Folder icon	Contain icon files for the app.
